# Assessing a penicillin allergy de-labelling implementation intervention in a UK hospital: a process evaluation reporting healthcare workers’ experiences

**DOI:** 10.1093/jacamr/dlaf174

**Published:** 2025-10-09

**Authors:** Neil Powell, Mathew Upton, Bridie Kent, Jonathan A T Sandoe, Sarah Tonkin-Crine

**Affiliations:** Pharmacy Department, Royal Cornwall Hospital, Truro TR1 3LJ, UK; School of Biomedical Sciences, University of Plymouth, Plymouth PL4 8AA, UK; School of Biomedical Sciences, University of Plymouth, Plymouth PL4 8AA, UK; School of Nursing and Midwifery, University of Plymouth, Plymouth, UK; Leeds Institute of Medical Research, University of Leeds, Leeds, UK; Nuffield Department of Primary Care Health Sciences, University of Oxford, Oxford, UK; National Institute for Health Research (NIHR) Health Protection Research Unit (HPRU) in Healthcare Associated Infections and Antimicrobial Resistance, University of Oxford, Oxford, UK

## Abstract

**Background and objectives:**

Penicillin allergy (penA) records prevent first-line penicillin antibiotic use, but more than 90% are incorrect after formal testing and can be removed (‘de-labelled’). We developed an implementation intervention package that supports a multi-professional non-allergy workforce to deliver penicillin allergy de-labelling (PADL) in a UK hospital. To explore the experiences of doctors, nurses, pharmacists and medicines optimization pharmacy technicians (MOPTs) of the implementation intervention package.

**Methods:**

Process evaluation utilizing semi-structured interviews with doctors, nurses, pharmacists and MOPTs with a target sample size of 20. Inductive reflexive thematic analysis was used to analyse the data.

**Results:**

Fifteen interviews were conducted between 7 November 2024 and 25 March 2025 with six doctors, five pharmacists and four MOPTs. PADL aligned well with the medicine’s reconciliation process, the process of accurately listing a person’s current medicines, which meant it better aligned with pharmacists’ and MOPTs’ roles than doctors’ roles. Healthcare worker (HCW) confidence to deliver PADL remained low among some doctors and pharmacists, but all reported that with time and support PADL would embed. Competing priorities in an inadequately resourced healthcare setting made PADL challenging. Professional bodies formally defining PADL as a core role for their HCWs would increase engagement with PADL. The PADL champion role was identified as key to the implementation of PADL.

**Conclusions:**

Competing priorities were limiting PADL engagement and as such PADL needs to be a core part of a HCW’s role for it to be prioritized. The champion is required to support PADL as a shared responsibility and needs to be available until the process is embedded into ways of working.

## Introduction

Up to 15% of hospitalized patients have a penicillin allergy (penA) record, which prevents first-line penicillin antibiotic use, potentially negatively impacting patient care, and is largely avoidable because more than 90% of penA records are incorrect.^1,2–4^

Due to the paucity of allergists in the UK, and elsewhere, non-allergy healthcare workers have explored, and demonstrated, the safety and effectiveness of non-allergist-delivered penicillin allergy de-labelling (PADL).^5^ Non-allergy healthcare workers (HCWs) are motivated to provide optimal patient care and view PADL as within their role if supported by hospital management and guidelines.^6–11^ However, many HCWs lack confidence to deliver PADL, viewing it as a complex task with resource implications and competing priorities, making it difficult to engage with PADL, in addition to the fear of causing patient harm.^6,7,9–14^ Several solutions have been proposed by HCWs that might increase PADL activity and include harnessing wider HCW support for PADL and ensuring trusted guidelines and protocols are available, as well as education and training.^9,14^ The PADL patient pathway requires ownership and leadership.^9^ It should be delivered collaboratively by multidisciplinary teams with a shared responsibility for delivering a simplified PADL procedure that is minimally disruptive on workload with integrated risk stratification tools and decision support integrated into electronic health records with increased barriers in place to access alternative antibiotics.^9,13,14,15^

We developed an implementation intervention package that supports a multi-professional non-allergy workforce to deliver PADL in a UK hospital.^15^ Implementation interventions are methods or techniques designed to change behaviours at organization, practitioner or patient levels to facilitate the adoption of a clinical intervention, in this case PADL.^16^ This study aimed to explore the experiences of doctors, nurses, pharmacists and medicines optimization pharmacy technicians (MOPTs) on the implementation intervention package and determine its acceptability, appropriateness and feasibility of delivering PADL.^15^ The findings will inform the further development and optimization of the PADL implementation intervention.^15^

## Methods

### Study design and setting

Process evaluation, utilizing semi-structured interviews, of an implementation intervention was tested with a multi-professional non-allergy workforce in a UK 760-bed district general hospital serving a population of 450 000 people. The hospital does not offer tertiary services, nor does it have an allergy department offering either inpatient or outpatient penicillin allergy de-labelling services.

### Implementation intervention

The PADL implementation intervention was launched on 10 June 2024 and is described elsewhere.^15^ In summary, the PADL patient pathway comprised a penA-focused history, risk assessment and, if assessed as low risk for genuine allergy, the patient was counselled on the risks and benefits of PADL before being offered de-labelling either on history alone or after a ward nurse administered a direct oral challenge (DOC) test using a penicillin antibiotic. If no symptoms were experienced within 1 h the patient was de-labelled.^15^ Permission was sought from senior responsible clinicians prior to DOC, but not for a direct de-labelling (DDL).

The implementation intervention comprised hospital PADL guidelines, patient leaflets to increase knowledge about incorrect penA labels and testing and highlight the benefits of being de-labelled, education and training for HCWs and named PADL champions.^15^ The PADL champion engaged with medical, surgical, nursing and pharmacy senior staff to raise awareness of PADL, to answer any questions about PADL, and to ensure senior-level support for PADL.

### Participant selection

We used purposive sampling to identify medical doctors, surgeons, nurses, pharmacists and MOPTs with a range of years’ experience to ensure a representative sample across all HCWs delivering PADL. Specialty lead consultants were e-mailed an invitation to participate in the study and requested to cascade the invitation to all doctors working in their specialty. Junior doctors [Foundation Year (FY), Core Trainee (CT) and Specialist Trainee (ST)] were invited via e-mail from the Medical Education Administrator. Clinical pharmacists and MOPTs were invited by e-mail via the pharmacy administrator. Clinical matrons were e-mailed the study invitation for cascade to ward nurses. Reminder e-mails were sent over the 5 month recruitment period and staff were approached by N.P. during clinical duties.

### Data collection

A semi-structured interview guide (available as Supplementary data at *JAC* Online) was developed based on relevant literature and informed by the Theoretical Domains Framework (TDF). The TDF is a framework enabling researchers to identify potential influences on HCW and patient behaviours related to implementation of evidence-based recommendations.^17^ HCWs’ experiences of the implementation intervention and their views on their role in delivering PADL were explored over Microsoft Teams, with audio recording, and transcription verbatim by an independent transcription services company. Written informed consent was collected.

### Data analysis

Data collection and analysis took place concurrently. The number of interviews and data quality were assessed according to information power and judged to be appropriate since this study’s aim was narrow and focused on a specific population in a single context. Transcripts were uploaded to NVivo 12. Inductive thematic analysis was used to analyse transcripts.^18^ N.P. familiarized himself with all transcripts before independently coding five transcripts. Codes were grouped into categories and these categories were named and refined through discussion with N.P. and S.T.C. The categories were used as a framework to code the remaining transcripts. The themes-generated additional codes were added as new data were identified and combined into original categories or used to add new categories. A reflexive approach with consideration of the study aims and researcher experiences and observations of delivery of the PADL intervention in the hospital was used to ensure contextualized understanding of the views of the interviewees. Once all data were coded, categories were grouped together to create themes representing the whole dataset.

## Ethics

This study was reviewed and approved by the Liverpool Central Research Ethics Committee (IRAS Project ID 299708).

## Results

### Participants

Fifteen interviews were conducted between 7 November 2024 and 25 March 2025, lasting 15–30 min (mean 22 min). Six doctors (two FY doctors; two doctors post FY training (middle grades) and two consultants (medical and surgeon), five pharmacists with mean 3.3 years’ experience (range 1–8 years) and four MOPTs with mean 18 years’ experience (range 0–36 years) were interviewed. Despite repeated e-mails and in-person communication with nurse leaders, nurse participants were not identified.

Three themes captured the views of HCWs.

### Theme 1: HCW awareness and engagement with PADL

#### Awareness of PADL pathway

The FY doctors, consultants and pharmacy staff were formally made aware of PADL through teaching and specialty meetings. Both middle-grade doctors had not formally been made aware of PADL, although were aware through colleague discussions. All interviewees, except for the two middle-grade doctors, had had some involvement in PADL.

*I've not had a go at removing the allergy because I wasn’t sure if anyone could, or if it was just the team that was doing the penicillin allergy de-labelling.* Junior doctor 3

#### Awareness of the risks and benefits of PADL

One consultant was able to cite several patient-specific benefits of PADL including better treatment of *Staphylococcus aureus* bacteraemia, but for others, the details of benefit were vague, and often uncertain.

Participants were unable to describe the risks of PADL but there was some concern about provoking anaphylaxis and about culpability.

*Well I suppose the only concern nagging in the back of your mind is what happens when a person does have an anaphylaxis.* Consultant 2

#### Engagement with PADL tools and training

The consultants and the FY doctors were aware of the PADL guidelines but had not accessed them. The middle grades were not aware of the PADL guidelines.

*I guessed there was a guideline because people were de-labelling and it feels like there was a potential risk to it, so doing it without a guideline seemed unlikely*. Middle-grade doctor 1

The penA history questions and the risk assessment tool were accessed routinely by MOPTs, whereas the patient information leaflets (PILs) were not; here participants reported that the PILs were less accessible.

*I keep them* [penA questions and risk assessment tool] *in the pharmacy report file*. Pharmacy technician 1

Pharmacists accessed the penA history questions and the risk assessment tool via the medicines reconciliation folder and via the guideline in the documents library on the hospital intranet, and these were described as comprehensive and easy to use because of the training provided and accessibility of the antimicrobial stewardship (AMS) pharmacists.

*I find that very useful* [decision support tool] *and I like the way it’s like colour-coded, I haven’t had the patient say they’ve had a reaction that hasn’t been on there.* Pharmacist 5

The FY doctors had received PADL training at their teaching sessions and one had received part 2 training. The training was described as a good foundation, enabling PADL engagement. The other doctors had not received formal PADL training. Delivering teaching to middle-grade doctors was a recognized challenge but including PADL in the regional specialty doctor training curriculum was suggested to reach doctors in training.

*The teaching behind it was actually a really good foundation for us to do something about* [PADL] *on the wards*. FY2 2

The MOPTs said that their training was adequate for their role taking penA histories, but further training was required to expand their role to DDL low-risk patients. Two MOPTs missed the formal training but were shown what was required by the ward pharmacists, enabling their engagement with PADL.

All pharmacists had completed the first part of the training and four of the five had completed part 2, which was described as adequate to prepare them for PADL. AMS pharmacist support facilitated ongoing learning. A junior doctor and a pharmacist suggested anaphylaxis training might give them more confidence with PADL.

[the training] *gave a good background of why we’re doing it and how to go about doing it, I was then able to start doing some of the histories.* Pharmacist 1

### Theme 2: Reasons for varying degrees of engagement with the PADL implementation intervention and suggested ways to increase engagement

#### Clinical workload, conflicting priorities and poor staffing

##### Doctors

The consultants said all doctors are juggling too many tasks. That, coupled with the lack of junior medical staff, made delivering PADL challenging, suggesting ward pharmacists were better placed to deliver PADL with doctors referring patients who might benefit, either via the hospital internal referral system, or to an outpatient clinic. FY doctors said competing priorities and poor staffing levels made prioritizing PADL difficult.

*It’s about competing demands and being chronically understaffed.* Consultant 1

*I think that, in different clinical settings, it’s quite difficult to act on it when there are other, maybe more pressing, clinical issues going on. If someone’s really unwell for example, it’s probably not going to be as much of a priority.* FY2 1

##### Pharmacists

Pharmacists described PADL as difficult to incorporate into their day given the other clinical priorities and unpredictability of daily workloads, but acknowledged that penA history-taking should be part of medicines reconciliation although the steps beyond that were challenging. One junior pharmacist said PADL was not a priority for their specialty lead pharmacist. As such, they felt their PADL efforts were not supported.

*PADL isn’t something highlighted as something that I should be doing* [by specialty lead pharmacist]. *They’d obviously rather I focused on getting all the patients done and doing their kind of work.* Pharmacist 5

The MOPTs all incorporated the structured penA history-taking into the medicines’ reconciliation process, which was described as easy to embed, and they reported doing it consistently.

#### PADL prompts/reminders and easier access to PADL tools may increase PADL engagement

A PADL template with the penA-focused questions within the electronic prescribing system with the tools required to implement PADL would streamline and encourage uptake, with prompts reminding HCWs to do the PADL assessment until complete.

One pharmacist said the AMS pharmacists had flagged potential PADL patients to them, which put these patients higher up their priority list.

*It’s helpful whenever* [the AMS] *team notify us of someone who’s a ‘Potential’ because then it’s brought to our attention—you can pull it up the list a bit—so that’s helpful.* Pharmacist 4

#### More HCW experience may increase PADL

One FY doctor said that they just need more experience with PADL. With time, continued education and further discussions with the AMS pharmacists, the FY doctor felt they would become more confident undertaking PADL. Several MOPTs said that the more penA histories they did and exposure they had to PADL the more confident they became.

*The more you do it, the easier it becomes.* Pharmacy technician 2

*With time, with education, with discussions, with you, I’ll get a pretty good grasp of what constitutes something high risk as opposed to medium, and what we can do about that* FY2 2

All the pharmacists said being supported with their first few PADL cases and practice with further patients meant they became more confident with time. Discussing cases with PADL champions provided the confidence needed. One pharmacist said that after a few months of starting PADL they saw firsthand the positive impact of PADL, which was impactful, whereas hearing about the positive impact of PADL on patients they didn’t know, did not resonate with them. Another pharmacist commented that with time the idea of PADL was becoming more widely accepted by HCWs and that PADL just needs time to embed.

*I do feel like it is a bit risky, but I think it would just take—just have to do it a few more times to feel more confident.* Pharmacist 5

### Theme 3: PADL alignment with HCW roles and clinical processes

#### PADL alignment with doctor roles

All the junior doctors said PADL aligned with their roles. However, engaging with PADL training and delivering PADL as part of routine practice did not align with any FY e-portfolio training objectives but did align with requirements in the e-portfolio beyond FY doctor years where PADL could be used as evidence for broadening their scope of practice.

Both consultants had experience of being asked permission to DOC their patients, which they agreed to, but otherwise PADL was outside their scope of practice. Delegating the task aligned with their role. When asked whether they thought it necessary to be asked permission prior to DOC, both felt that, as senior responsible clinicians, it was right they were asked.

*I mean, I think it’s nice to be asked the question because ultimately I’m responsible for that patient’s care and so if there is a complication, I sort of feel duty bound to be looking after them and to manage that appropriately afterwards. So I think it’s reasonable to ask the consultant in charge of that patient if that’s appropriate.* Consultant 2

#### PADL alignment with pharmacist roles

PADL was described by pharmacists as a medicines optimization intervention that aligns with pharmacist roles. The penA history-taking was described as something that should be part of standard care, but the other stages needed training and education to enact. Obtaining patient consent prior to de-labelling was described as a new skill for pharmacists, particularly the non-medical prescribers. Knowing whether the patient had understood the information and therefore given informed consent to de-label caused some concern. One pharmacist thought PADL was a great way to demonstrate their value to the multi-disciplinary team (MDT), adding to pharmacist roles and making them feel useful, positively impacting antibiotic stewardship.

*And it’s quite a good like way for pharmacists to like show* [pharmacist] *value* [to the MDT]. Pharmacist 5

*Informed consent is not something that you do as a pharmacist until you’re hitting the point where you’re doing the prescribing. It’s not our bread and butter.* Pharmacist 2

#### PADL alignment with MOPT roles

MOPTs said they viewed PADL as an important part of their role. PenA history-taking was described as straightforward and it was quick to normalize into the medicines reconciliation process. Although examples of MOPTs undertaking DDL were infrequent, MOPTs described DDL of low-risk patients as within their remit.

*That part of it* [penA history-taking] *is pretty straightforward to be fair. Obviously if it was going to go a bit more in-depth then probably we might need a little bit of support from the ward pharmacist.* Pharmacy technician 1

*I wouldn’t have a problem with it the basic de-labelling. Because I think we are capable of making that decision.* Pharmacy technician 2

#### PADL alignment with clinical processes

Both consultants said the best way to refer inpatients to PADL would be to use the mechanisms for patient referral that already exist. One FY doctor said the PADL process was not accessible enough for busy ward doctors, with too many steps that need actioning and suggesting streamlining the process.

*I’ll be honest with you, it’s quite heavy. It doesn’t sound that heavy, but when you have a job list to get through, your mind is not in a position to be thinking, ‘Oh, I need to tick this box, I need to send this to the GP’. You know, grab the e-mail like, I’ll tell you right now, don’t think people are going to do that. It just needs to be a bit more user friendly like just mention it on the discharge summary, have the GP action it, tick box. Yeah, just a little bit more accessible.* FY2 2

The MOPTs said that penA history-taking aligns well with the medicines reconciliation process and although it takes a little longer, it doesn’t significantly impact their work rate. Pharmacists said penA history-taking aligns with the medicines reconciliation process and is now part of normal practice. One pharmacist said it was the subsequent steps that negatively impact on workload.

*It’s become normal practice now. Doing the history—it’s just like an extra stage—if they’ve got the penicillin allergy. I think it flows well into a normal history-taking—and the questions are clear.* Pharmacist 4

#### Well supported to deliver PADL

MOPTs said they felt well supported by the ward pharmacists and AMS pharmacists to deliver PADL. Several pharmacists said AMS pharmacist support is all that they require to organize a DOC and that it was reassuring having support available, particularly for the first few PADLs. Some pharmacists would not feel confident de-labelling without AMS pharmacist support.

*I feel like you have supported us and having you guys there to just like run through a case. I think that’s like pretty much all the support that you would need really.* Pharmacist 5

One consultant said that their trust in the AMS lead and their confidence in their demonstrated decision-making meant that they were not concerned about safety and were supportive of PADL.

## Discussion

### Main findings

Awareness of the PADL implementation strategy was high except for middle-grade doctors who were not reached by awareness-raising activities, which limited their engagement with PADL.

PADL aligned well with the medicines reconciliation process, well established in UK hospitals, which meant it better aligned with pharmacist and MOPT roles than doctors’ roles.^19^ It was easy to make the tools accessible to pharmacy staff but less practical to make the tools accessible to doctors.

HCW confidence to deliver PADL remained low among some doctors and pharmacists, but all reported that, with time and support, their confidence would build and PADL would embed. Competing priorities in an inadequately resourced healthcare setting made PADL challenging. PADL is not currently defined as a role for non-allergy HCWs in the UK, remaining a core role for allergists.^20,21^ Professional bodies for the healthcare professions formally defining PADL as a core role for HCWs in their professions would make PADL more a priority.

The AMS champion role was identified as key to the implementation of PADL and was utilized by HCWs to discuss cases and deliver PADL. The reliance on champions waned as HCWs became more confident. The PADL champion was trusted, which facilitated the garnering of senior clinician support for PADL.

### Comparison with the literature

Several studies have reported clinician concerns about the safety of PADL, although the majority of clinicians acknowledged that the benefits outweighed the risks and found that providing optimal care motivated HCWs to deliver PADL.^9–12,14^ Hanssen *et al*. found that clinicians felt the risks outweighed the benefits but in their intervention no training was provided to HCWs.^12^ We found some concerns about the risks of PADL but they were not described as outweighing the benefits, which may be due to the local awareness-raising and training.

Competing priorities are commonly reported as a barrier to PADL.^10,11,13^ In the study by Ngassa *et al.*,^10^ clinicians reported operational barriers to PADL, suggesting it to be low priority. Alagoz *et al.*^11^ reported pharmacists’, hospitalists’ and specialty consult services’ views that workload and competing priorities prevented implementation of penicillin allergy protocols in the inpatient setting, with PADL hindered by the need to prioritize other duties. A study by Gray *et al.*^13^ reported physicians’, nurses’ and pharmacists’ views on PADL, where a lack of time to evaluate β-lactam allergies relative to higher-priority tasks was presented as a barrier to PADL. In the study by Gray *et al.*, pharmacists and pharmacy technicians were identified as best placed to deliver PADL due to their medicines role, their skills and proven track record with safe delivery of PADL.^13^ Pharmacists were identified in our study as perhaps best placed to deliver PADL but that it was a shared responsibility.

Alagoz *et al.* reported that doctors and pharmacists lacked confidence with PADL, needing frequent practice and training.^11^ We found low confidence at the beginning of PADL implementation. The training delivered in our hospital was both a theory-based lecture and case-based (simulation), which respondents said gave them the confidence needed to start de-labelling and that with time and practice confidence grew but the support from the AMS pharmacist PADL champions was identified as key to the sustained confidence building of staff. Refresher training may also be required to build and maintain confidence. Alagoz *et al.* reported that clinicians lacked knowledge of where the PADL tools were located, but that once found, junior doctors and pharmacists reported that the algorithms were easy to follow, which is similar to our findings.^11^

### Implications for practice

The requirement for PADL champions to lead PADL initiatives is apparent from our study and the wider literature.^8,9,11,13,14^ PADL champions are needed to lead the PADL initiative, to raise awareness, to educate and to train HCWs and to support HCWs delivering PADL, providing reassurance and continued support with decision-making, when required. Also, ensuring wider clinical team support for PADL is required to give junior doctors and pharmacists confidence and to motivate them to engage with PADL. This creates an environment of shared responsibility for PADL decisions, particularly whilst the culture around PADL is changing.

With training, we found that doctors, pharmacists and MOPTs were able to engage with PADL. Whilst for MOPTs this training was enough to embed the penicillin history-taking into their role, it wasn’t the same for doctors and pharmacists. Pharmacists and junior doctors need further PADL training and ongoing access to a PADL expert/lead for support for them to gain confidence and to further embed PADL into their routine ward work. This alone is unlikely to result in sustained PADL activity without prompts from other HCWs and/or the electronic prescribing system. Both the pharmacists and the junior doctors need to gain confidence over time, which means that embedding PADL is likely to be a time-dependent process. Consultants are least likely to engage with de-labelling patients, and setting up a referral system for patients is the best way to engage senior doctors with the PADL process, their role being to prompt PADL delivered by others.

Ensuring PADL is defined within pharmacists’ and MOPTs’ job roles/scope of practice, either locally or nationally, will be necessary. Ensuring the AMS pharmacist can champion PADL and provide ward pharmacist and MOPT education, training and ongoing support may allow PADL to embed over time.

### Strengths and limitations

We interviewed a range of HCWs involved in the PADL process with varying experience and working across several adult inpatient areas but recognize the findings may not be generalizable due to the single centre the HCWs were recruited from.

We were unable to interview nurses, which limits our understanding of their views on their role in the PADL process, namely the pre- and post-DOC monitoring and administration of the DOC dose. Anecdotally, the AMS pharmacist did not come across any reluctance from nursing staff to administer DOC doses or monitor patients.

### Conclusions

We found that in our hospital the interviewed HCWs had relevant skills and knowledge and therefore could deliver PADL, which in our setting aligned best with the medicines reconciliation process undertaken by pharmacy staff, although senior doctors wanted to retain involvement in decision-making. We found competing priorities were limiting PADL engagement and as such PADL needs to be a core part of HCWs’ roles for it to be prioritized. In our setting we found that a champion, or some structure, is needed to support shared responsibility when people are asked to do this for the first few times, and needs to be available until the process is embedded into ways of working.

## Supplementary Material

dlaf174_Supplementary_Data

## Supplementary data

The semi-structured interview guide is available as Supplementary data at *JAC* Online.
