# Supplementary material for: Assessing a penicillin allergy de-labelling implementation intervention in a UK hospital: a process evaluation reporting healthcare workers’ experiences
Source: JAC Antimicrob Resist. 2025 Oct 9;7(5):dlaf174. doi: 10.1093/jacamr/dlaf174 (PMC12509609; doi:10.1093/jacamr/dlaf174)
Supplement: dlaf174_Supplementary_Data [file dlaf174_supplementary_data.docx]

**Removing Erroneous Penicillin Allergy Labels (REPeAL)**

**Interview Topics Overview (Health Care Professionals)**

**INTRODUCTION:**

Thank you for taking the time to speak to me today. My name is Neil. I am a consultant antimicrobial pharmacist undertaking a research project at the Royal Cornwall Hospital Trust.

I want to explore healthcare worker views and experiences of delivering the penicillin allergy de-label patient pathway. I will describe this briefly in a moment. I am interested in finding out ways the delivery of this pathway can be improved. I would like to hear about your experiences and your opinions, in your own words, there are no right or wrong answers.

If you need to stop the interview, then please feel free to say so at any time. Do you have any questions before we get started?

**Objectives**

Describe PADL to participant using the bullet points below & the PowerPoint slide;

- A risk stratification algorithm is often used to enable healthcare professionals to risk stratify patients into low or high risk of future reactions.
- Depending on the low-risk symptoms, patients can be offered a ‘direct de-label’ (with the patient’s consent the allergy record can be removed on history alone) or a direct oral challenge test (single dose of amoxicillin 500mg or index penicillin and observed for 60 minutes).
- Low risk patients include patients without evidence of IgE mediated reactions or evidence of severe delayed reactions Low risk patients and include those with a benign delayed rash
- Communicate results to the GP and others caring for the patient.

********************** Start recording & check consent form signed ************************

1. Can you tell me what you know about adult inpatient penicillin allergy de-labelling? Do you think your colleagues are aware of PADL and the resources?
2. Can you tell me about your experience of PADL?

Prompts: How do you take a penicillin allergy history? What tools have you used to help with this (e.g. hospital guideline, risk assessment tool)? What experience do you have of de-labelling patients?

1. Please tell me which resources you have accessed and what you found useful? What did you think of the guideline? could they access it easily, what information was useful. How did the risk stratification tool apply to their patients? What did they have problems with? Who did they ask for help? What was their experience of discussing with patients? What did patients think of PADL? How did they change the medical record? How did they write to the GP? What did they have to amend and do differently in their context?
2. How important do you think PADL is in your role?

Prompts: or ward or hospital?

1. Have you had any difficulties in engaging with PADL or using tools available?
2. Which parts of the pathway are difficult for you to put into practice? E.g. EPMA penA update, contacting GPs to update penA status?)
3. From your experiences so far with PADL, how do you think HCPs could be better supported to deliver PADL? What would make it easier for you to follow this pathway?
4. Which behaviours in the penicillin allergy de-label patient pathway do you feel more familiar with? What helped you become more familiar? How long have you been familiar (i.e. since REPEAL)?

Prompts: how familiar are you with the behaviours required to deliver the PADL pathway? Which behaviours align with your role, and you are more familiar with?

1. Which behaviours in the penicillin allergy de-label patient pathway do you feel less familiar undertaking? What would help you become more competent?

Prompts: Which behaviours align less well with your role or patient flow?

1. How do you feel about being asked to do this as part of your role?

Prompts: is this relevant to your role?

1. Have you identified any training needs or require any further support to enable you to carry out PADL with your patients?
2. What are your concerns with the PADL pathway?

Prompts: what does not work about this approach in your setting?

1. How could your organisation support this way of working? How do you think this approach could be better incorporated into the organisation?

Prompts: what factors would help introduce this new way of working? What factors would hinder this new way of working?

1. Is there anything else you’d like to add?

- What is your profession?
- How many years have you worked as a nurse/doctor/pharmacist/?
